# Transjugular Retrograde Obliteration prior to Liver Resection for Hepatocellular Carcinoma Associated with Hyperammonemia due to Spontaneous Portosystemic Shunt

**DOI:** 10.1155/2013/809543

**Published:** 2013-08-19

**Authors:** Fumio Chikamori, Nobutoshi Kuniyoshi

**Affiliations:** ^1^Department of Surgery, Kuniyoshi Hospital, 1-3-4 Kamimachi, Kochi City, Kochi 780-0901, Japan; ^2^Department of Internal Medicine, Kuniyoshi Hospital, 1-3-4 Kamimachi, Kochi City, Kochi 780-0901, Japan

## Abstract

A 67-year-old woman had hepatocellular carcinoma (HCC) measuring 3.7 cm at S8 of the liver with hyperammonemia due to a spontaneous giant mesocaval shunt. Admission laboratory data revealed albumin, 2.9 g/dL; total bilirubin, 1.3 mg/dL; plasma ammonia level (NH_3_), 152 g/dL; total bile acid (TBA) 108.5 *μ*moL/L; indocyanine green retention rate at 15 min (ICG15), 63%. Superior mesenteric arterial portography revealed a hepatofugal giant mesocaval shunt, and the portal vein was not visualized. Before surgery, transjugular retrograde obliteration (TJO) for the mesocaval shunt was attempted to normalize the portal blood flow. Via the right internal jugular vein, a 6 F occlusive balloon catheter was inserted superselectively into the mesocaval shunt. The mesocaval shunt was successfully embolized using absolute ethanol and a 50% glucose solution. Eleven days after TJO, NH_3_, TBA, and ICG15 decreased to 56, 44, and 33, respectively. Superior mesenteric arterial portography after TJO revealed a hepatopetal portal flow. Partial hepatectomy of S8 was performed 25 days after TJO. The subsequent clinical course showed no complications, and the woman was discharged on postoperative day 14. We conclude that the combined therapy of surgery and TJO is an effective means of treating HCC with hyperammonemia due to a spontaneous portosystemic shunt.

## 1. Introduction

Hepatocellular carcinoma (HCC) with hyperammonemia due to a spontaneous portosystemic shunt (PSS) is not common, and the guidelines for such a condition have not been established yet [1]. Liver function is an important factor to determine the treatment strategy for HCC.

To lower morbidity after hepatic resection, the Makuuchi criteria, including the presence or absence of ascites, serum total bilirubin level, and the plasma indocyanine green retention rate at 15 min (ICG15), are widely used [2, 3]. However, the existence of PSS often increases the level of ICG15 and the plasma ammonia level (NH_3_) and reduces the hepatopetal portal blood flow. We previously reported that transjugular retrograde obliteration (TJO) for PSS reduced ICG15 and NH_3_ [4]. A mesocaval shunt is one of the PSSs. Here, we describe a case of HCC associated with hyperammonemia due to a spontaneous mesocaval shunt treated by the combined therapy of surgery and TJO.

## 2. Case Report

 A 67-year-old woman suffered from HCC with hyperammonemia due to a spontaneous giant mesocaval shunt. Six months before that, she had undergone interferon therapy for hepatitis C. However, follow-up CT examination revealed HCC, so she was referred to our department for further evaluation and treatment.

**Figure 1:**
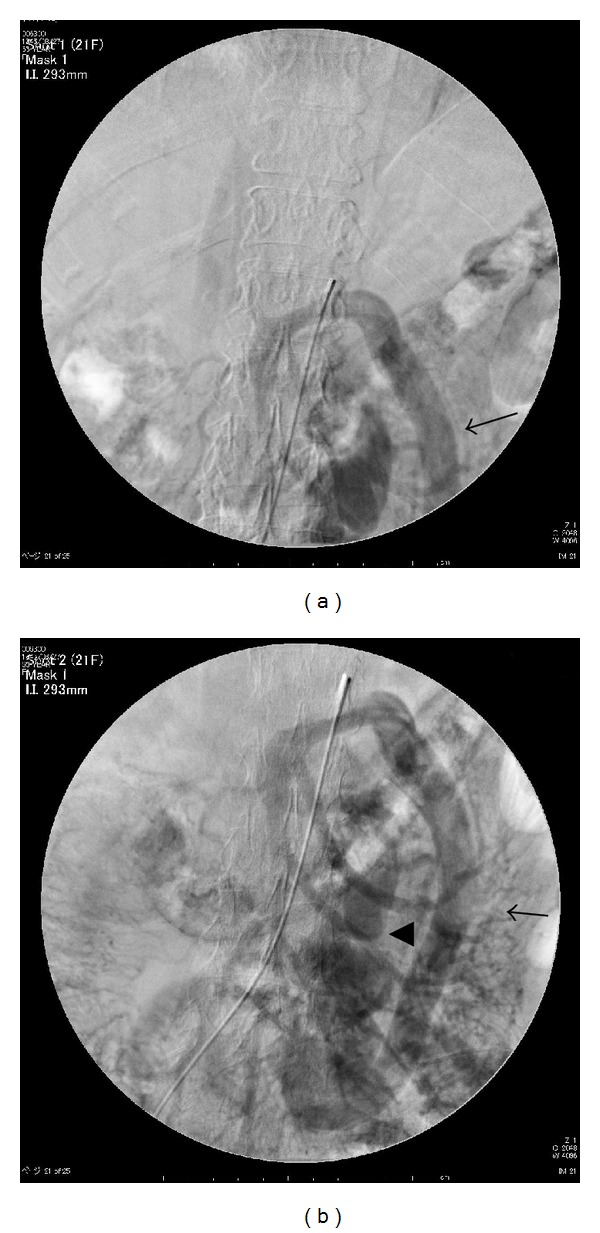
(a) Superior mesenteric arterial portogram shows hepatofugal giant mesocaval shunt (arrow). Portal vein was not visualized. (b) Superior mesenteric arterial portogram: mesenteric venous blood was drained into the inferior vena cava via dilated inferior mesenteric vein (arrow), left ovarian vein (arrowhead) and left renal vein.

On admission, her vital signs were stable. The patient was conscious and alert. Her palpebral conjunctivae were pale. Admission laboratory data were as follows: white blood cell count, 4300/*μ*L; hemoglobin, 10.6 g/dL; platelets, 187000/*μ*L; albumin, 2.9 g/dL; total bilirubin, 1.3 mg/dL; aspartate aminotransferase, 46 IU/L; alanine aminotransferase, 14 IU/L; cholinesterase, 132 U/L; prothrombin time, 62%; hepaplastin test, 62%: NH_3_, 152 g/dL; total bile acid (TBA), 108.5 *μ*moL/L: ICG15, 63%. Tumor marker levels were as follows: alpha-fetoprotein (AFP), 88.5 nG/mL; protein induced by vitamin K absence or antagonist II (PIVKA-II), 5130 mAU/mL. Hepatic function was classified as Child-Pugh class A (score 6). Hepatitis B surface antigen was negative. Hepatitis C virus (HCV) antibody was positive, but HCV-RNA was not detected. Ultrasonography and contrast enhanced computed tomography revealed HCC measuring 3.7 cm in diameter at S8 of the liver and a markedly tortuous mesocaval shunt. An endoscopic examination revealed no esophagogastric varices. Superior mesenteric arterial portography revealed a hepatofugal giant mesocaval shunt, and the portal vein was not visualized (Figures 1(a) and 1(b)).

**Figure 2:**
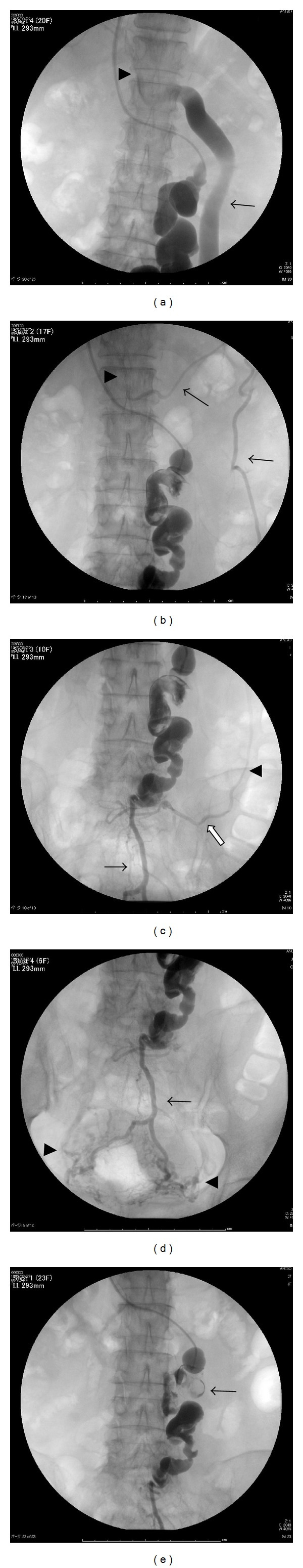
(a) Transjugular retrograde obliteration (TJO) on the 1st day: retrograde shunt venogram shows left ovarian vein and dilated inferior mesenteric vein (arrow) and portal vein (arrowhead). (b) Retrograde shunt venogram on the 2nd day shows marginal vein (arrow) communicated with portal vein (arrowhead). (c) Retrograde shunt venogram on the 2nd day shows superior rectal (arrow), sigmoid (white arrow), and marginal veins (arrowhead). (d) Retrograde shunt venogram on the 2nd day shows superior rectal vein (arrow) communicated with bilateral internal iliac veins (arrowhead). (e) Retrograde shunt venogram on the 3rd day shows thrombus formation (arrowhead) in the mesocaval shunt. The marginal vein communicated with portal vein was not visualized.

Before surgery, TJO for the mesocaval shunt was attempted to normalize the portal blood flow, NH_3_, and ICG15. Via the right internal jugular vein, an 8 F cobra shaped sheath was inserted into the left renal vein. Then, a 6 F occlusive balloon catheter was inserted superselectively into the mesocaval shunt (Figure 2(a)). On the 1st day of TJO, 8 mL of absolute ethanol and 100 mL of a 50% glucose solution were injected into the mesocaval shunt intermittently.

On the 2nd day, the marginal vein which communicated with the portal vein and the superior rectal vein which communicated with the bilateral internal iliac veins were revealed, so 1 mL of absolute ethanol and 20 mL of a 50% glucose solution were injected again (Figures 2(b), 2(c), and 2(d)). On the 3rd day, the marginal vein was not visualized. After confirming the thrombus formation in the mesocaval shunt, the catheter was removed (Figure 2(e)).

**Figure 3:**
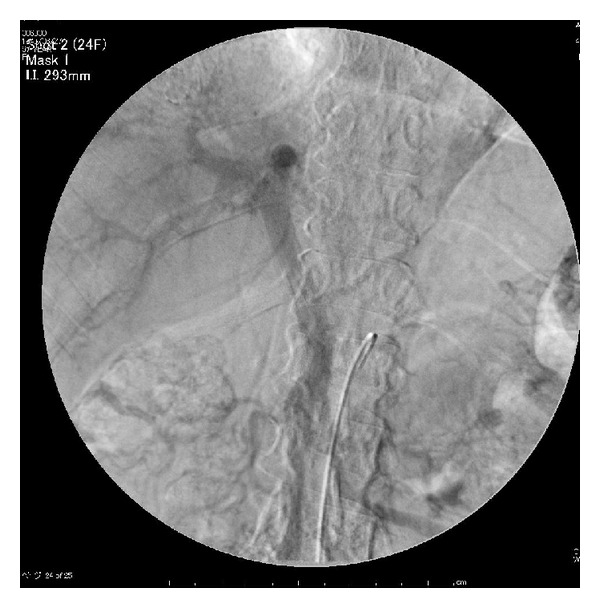
Superior mesenteric arterial portogram after TJO shows hepatopetal portal blood flow.

Eleven days after TJO, NH_3_, TBA, and ICG15 decreased to 56, 44, and 33, respectively. Superior mesenteric arterial portography after TJO revealed a hepatopetal portal blood flow (Figure 3). Partial hepatectomy of S8 was performed 25 days after TJO. The subsequent clinical course showed no complications, and the woman was discharged on postoperative day 14. AFP/PIVKA-II levels 2 and 14 weeks after surgery decreased to 21.2/79 and 3.7/24, respectively. Follow-up examinations for 6 months after the combined therapy have not indicated any recurrence of HCC or hyperammonemia.

## 3. Discussion

We successfully treated HCC with hyperammonemia due to a spontaneous PSS by the combined therapy of surgery and TJO. HCC with hyperammonemia is an uncommon condition. Hepatic failure is the most lethal complication of hepatectomy. Insufficient portal blood flow is one of the causes of hepatic failure [5]. The treatment for such a condition has not yet been established. Hepatectomy with simultaneous ligation of spontaneous PSS has been reported [6–8]. However, hepatectomy for patients with poor liver function is invasive, so we have to evaluate preoperative liver function precisely. If liver function is modified by abnormal portal blood flow, we have to reevaluate it after normalization of portal blood flow.

We applied the interventional radiology technique (IVR) to normalize the portal blood flow. Vascular anatomy is important for treating PSS by IVR. A mesocaval shunt is one of the PSSs, and it is supplied by the inferior mesenteric vein and drained by the gonadal or renal vein. In addition, we have to recognize that the shunt communicates with superior rectal and sigmoid veins. There are three IVR approaches for mesocaval shunt obliteration. Percutaneous transhepatic obliteration (PTO) [9, 10] and transileocolic vein obliteration (TIO) [11] are embolization techniques via the blood supply route. Retrograde transvenous obliteration (RTO) is an embolization technique via the blood drainage route. PTO for varices is occasionally used for acute variceal hemorrhage; however, it has the risk of intraperitoneal bleeding and is invasive. Minilaparotomy is necessary for TIO. Therefore, we chose RTO in the present case. 

There are only a few reports of combined therapy using surgery and RTO for HCC with PSS [12, 13]. RTO such as TJO [14] or balloon-occluded transvenous obliteration (B-RTO) [15] is popular in Japan for gastric variceal treatment. TJO was reported as a transjugular approach, which maintains the balloon catheter for 24 hours, and B-RTO was reported as a transfemoral approach, which maintains the catheter for 30 minutes originally [14, 15]. TJO has an advantage over the femoral approach in being able to obliterate the shunt superselectively. Superselective obliteration can reduce the volume of sclerosant required for shunt obliteration.

We already confirmed that TJO for chronic portosystemic encephalopathy reduced NH_3_ and ICG15 [3]. In the present case, we applied TJO for the mesocaval shunt before hepatectomy to normalize the portal blood flow during which we should pay attention to the communicating routes of the mesocaval shunt, which seem to be a giant solitary line on a superior mesenteric arterial portogram. Actually, it is not solitary and communicates with the superior rectal and sigmoid veins. A retrograde shunt venogram on the 2nd day revealed these communicating routes. The superior rectal vein communicates with the bilateral iliac veins, which lead to systemic circulation, but the sigmoid vein communicates with the marginal vein which leads to portal circulation. The most important issue is how to disconnect the mesocaval shunt from the portal circulation completely. Therefore, we had to reinject the sclerosant on the 2nd day. We could confirm that the marginal vein was not visualized by retrograde shunt venography on the 3rd day. TJO contributed to the protecting portal blood steal after hepatectomy, and the patient's postoperative course was uneventful. We conclude that the combined therapy of surgery and TJO is an effective means of treating HCC with hyperammonemia due to a spontaneous PSS.
